# Antibiotic Resistance and Mechanisms of Pathogenic Bacteria in Tubo-Ovarian Abscess

**DOI:** 10.3389/fcimb.2022.958210

**Published:** 2022-07-27

**Authors:** Huanna Tang, Hui Zhou, Runju Zhang

**Affiliations:** ^1^ Women’s Reproductive Health Research Key Laboratory of Zhejiang Province and Department of Reproductive Endocrinology, Women’s Hospital, Zhejiang University School of Medicine, Hangzhou, China; ^2^ Department of Infectious Disease, Second Affiliated Hospital, Zhejiang University School of Medicine, Hangzhou, China

**Keywords:** tubo-ovarian abscess, antibiotic resistance, *Escherichia coli*, *Bacteroides fragilis*, gram-positive anaerobic cocci, antibiotics

## Abstract

A tubo-ovarian abscess (TOA) is a common type of inflammatory lump in clinical practice. TOA is an important, life-threatening disease, and it has become more common in recent years, posing a major health risk to women. Broad-spectrum antimicrobial agents are necessary to cover the most likely pathogens because the pathogens that cause TOA are polymicrobial. However, the response rate of antibiotic treatment is about 70%, whereas one-third of patients have poor clinical consequences and they require drainage or surgery. Rising antimicrobial resistance serves as a significant reason for the unsatisfactory medical outcomes. It is important to study the antibiotic resistance mechanism of TOA pathogens in solving the problems of multi-drug resistant strains. This paper focuses on the most common pathogenic bacteria isolated from TOA specimens and discusses the emerging trends and epidemiology of resistant *Escherichia coli*, *Bacteroides fragilis*, and gram-positive anaerobic cocci. Besides that, new methods that aim to solve the antibiotic resistance of related pathogens are discussed, such as CRISPR, nanoparticles, bacteriophages, antimicrobial peptides, and pathogen-specific monoclonal antibodies. Through this review, we hope to reveal the current situation of antibiotic resistance of common TOA pathogens, relevant mechanisms, and possible antibacterial strategies, providing references for the clinical treatment of drug-resistant pathogens.

## 1 Introduction

Pelvic inflammatory disease (PID) is a polymicrobial infection of the female upper genital tract with an estimated prevalence of 3.6–10% among reproductive-age women (Kreisel et al., 2021). More specifically, we are concerned about the fact that women with PID will develop long-term reproductive sequelae such as persistent pelvic pain, infertility, and ectopic pregnancy. Studies show that 2.3–20% of hospitalized PID cases have developed into tubo-ovarian abscess (TOA), which can be a life-threatening condition given the potential of abscess rupture and sepsis (Mollen et al., 2006; Kairys and Roepke, 2022). The mortality rate of TOA was once as high as 12% when the abscess was not drained promptly on time in the mid-20th century (Vermeeren and Te Linde, 1954).

With the development of effective antibiotics and prompt surgical intervention, TOA-related mortality is surprisingly low, with a rate of one death per 740 women in 2019 (Shigemi et al., 2019). Ginsburg and colleagues followed 160 patients with TOA for several years; of the 120 patients in whom the reproductive function was preserved, only 7.5% of the women reported pregnancy (Ginsburg et al., 1980). More than 85% of infections in PID patients are caused by sexually transmitted diseases or vaginitis-related pathogens, with the remaining infections being caused by respiratory or intestinal germs that colonize the lower tract (Brunham et al., 2015). However, the causative agents identified in TOA patients are not the same as those isolated from PID cases without TOA. The most prevalent isolates in TOA include *Escherichia coli* (*E. coli*, 37%), *Bacteroides fragilis* (*B. fragilis*, 22%), other *Bacteroides* (26%), *Peptostreptococcus* (18%), and *Peptococcus* (11%) (Landers and Sweet, 1983). Surprisingly, the main pathogens of PID, *Neisseria gonorrhoeae* and *Chlamydia trachomatis*, are rarely detected in clinical TOA specimens, with 3.8% positive for *Neisseria gonorrhoeae* and no report for *Chlamydia trachomatis* (Krivak et al., 2004).

Broad-spectrum antibiotic therapy, should be started as soon as possible to prevent the worrying consequences of TOA. The response rate of antibiotic treatment is about 70%, whereas drainage or surgical intervention is necessary in refractory cases (Akselim et al., 2021). Antibiotics are chosen based on the most prevalent causative agents of TOA and their ability to penetrate the abscess cavity. The optical medication is debatable for different combinations of antibiotics have been used worldwide which manifest geographical variation (Long and April, 2017; Savaris et al., 2017). Approximately 30 different antibiotics from eight subclasses are prescribed in the clinic. The Centers for Disease Control and Prevention recommendations for the parenteral treatment of moderate to severe PID with TOA include cefotetan plus doxycycline, cefoxitin plus doxycycline, and clindamycin plus gentamicin. Clindamycin and metronidazole are preferred for their anaerobic spectrum of action and efficacy in penetrating the abscess wall (Kairys and Roepke, 2022). Based on the findings, no single medicine or combination of drugs appears to be better than the others. It is vital to remember that, while antibiotics are necessary for the treatment of TOA, they are rarely enough (Karaca et al., 2018; Chan et al., 2019). The long-term irrational use of antibiotics promotes the development of diverse resistance genes in pathogenic organisms, which reduces the efficacy of antibiotics and increases the odds of surgical intervention of TOA (Goharkhay et al., 2007; Fouks et al., 2019).

Health professionals have to rely on PID data sources for the empirical use of antibiotic agents for the surveillance data on the antibiotic resistance of TOA are lacking. Thorough understanding of pathogenic spectrum, drug-resistant conditions, and appropriate choice of antibiotic therapy prompt a good response to treatment. The majority of the evidence for PID comes from older researches that may not represent current changes in antibiotic sensitivity patterns or newer diagnostic techniques, especially for severe PID with TOA. To better guide clinical therapy toward TOA, this article reviews the function of common antibiotic resistance genes and their mediated antibiotic resistance mechanisms in typical TOA-related pathogens. We also discuss some novel methodologies that could provide therapy alternatives in the near future. While these strategies are still in the early stages of development and their efficacy is unknown, they have the potential to alter severe TOA management in the field of multi-drug resistance.

## 2 Antibiotic resistance of several prominent TOA pathogens

### 2.1 E. Coli

#### 2.1.1 Antibiotic Resistance of *E. Coli*

*E. coli*, the most prevalent Gram-negative bacterial pathogen, is well established as the causative agent of intestinal infections. Additionally, *E. coli* is the most common enteric Gram-negative bacillus found in women’s genital tracts, causing vaginal and/or endocervical colonization as well as various infections, including intra-amniotic, puerperal, and neonatal infections. *E. coli* has also been regarded as the most frequent bacteria isolated from the upper genital tract in patients with TOA (Schindlbeck et al., 2014). Antibiotics for *E. coli* have been applied in human and veterinary treatment for a long time, causing antimicrobial resistance to reach a serious situation. Obtaining sequential data on resistance and multidrug resistance from *E. coli* strains isolated from TOA is difficult. Recent studies from the United States show high resistance to β-lactams antibiotics (50.6%), trimethoprim/sulfamethoxazole (27.3%), fluoroquinolones (25.7%), and nitrofurantoin (56% each) in *E. coli* (Dunne et al., 2022). Among the 134 *E. coli* isolates collected from Hangzhou nosocomial bloodstream infections from 2013 to 2016, approximately 41.5% were identified as extended-spectrum β-lactamase (ESBL)-producing-positive by Jiang et al. (2019). Even though medical institutions have imposed strict controls and rules on the use of antibiotics, publications suggest that the growing trend of drug resistance in *E. coli* continues (Aliabadi et al., 2021).

#### 2.1.2 The Antibiotic Resistance Mechanism of *E. Coli*

Multidrug-resistant *E. coli* has become one of the most common pathogens of the upper vaginal tract, posing danger to women’s health across the world. The resistance mechanisms of *E. coli* to different types of antibiotics are as follows: (1) production of hydrolase or modified enzyme to inactivate the drugs, (2) changes in the target site of the antibiotics, (3) bacterial cell membrane permeability changes (such as reduction of porins and formation of biofilms), and (4) enhancement of bacterial active efflux pumps that prevent antibiotics from reaching effective bactericidal concentrations (**Table 1**).

**Table 1:** The main mechanism of drug resistance in *E. coli*.

Mechanism	Related research	Related drugs
Production of hydrolase or modified enzyme	ESBL: TEM, SHV, and CTX-M (Mulder et al., 2021) AmpCs (Froding et al., 2020) Carbapenemases: KPC, NDM, VIM, OXA, and IMP (Han et al., 2020) Aminoglycoside-modifying enzyme: AAC, ANT, APH	Penicillins, cephalosporins, fluoroquinolones, some animoglycosides, and TMP-SMX Carbapenem Animoglycosides
Target site mutation	gene *gyrA*, *gyrB*, *gyrC*, mutation (Hastak et al., 2020)	Quinolones and fluoroquinolones
Decreased cell permeability or active efflux pump	ABC, MSF, RND, MATE, and SMR (Nolivos et al., 2019)	Tetracycline, chloramphenicol, β-lactam, quinolone, *etc*.

TMP-SMX, trimethoprim-sulfamethoxazole.

One of the primary causes of antibiotic resistance in *E. coli* is the production of β-lactamase enzymes, causing resistance against many broad-spectrum β-lactams, which are first-line drugs in the treatment of PID. ESBLs, as a particularly troublesome group of β-lactamases (including TEM, SHV, and CTX-M types), have the capacity to hydrolyze penicillins and first-, second-, and third-generation cephalosporins. ESBLs are inhibited by b-lactamase inhibitors such as clavulanic acid, sulbactam, or tazobactam. AmpC β-lactamases are another source of drug resistance in *E. coli*. AmpC is resistant to penicillins, first-, second-, and third-generation cephalosporins, and cephamycins and are not inhibited by clavulanic acid (Poirel et al., 2018). Carbapenems have become empiric choices when ESBLs or AmpC-lactamases turn into concerns. Unfortunately, the emergence of carbapenemase (KPC, NDM, VIM, OXA, and IMP) makes the treatment more difficult (Han et al., 2020). Aminoglycosides are frequently used to treat Gram-negative bacteria, including *E. coli* infections. However, resistance to aminoglycosides was found in 79.5% of the *E. coli* isolates (Ojdana et al., 2018). The degree of resistance was so high that they were rendered practically worthless in some cases. One of the most common mechanisms of resistance to aminoglycosides among *E. coli* is the production of aminoglycoside-modifying enzymes (AMEs), which are divided into three families: aminoglycoside acetyltransferases, aminoglycoside nucleotidyltransferases, and aminoglycoside phosphoryl transferases (Ghotaslou et al., 2017). AMEs act on the specific amino group or hydroxyl group of the antibiotics, making the affinity between antibiotics and the binding site of bacterial ribosome decrease.

Quinolones and fluoroquinolones are important broad-spectrum antibacterial medicines used to treat a variety of infections in gynecology. In *E. coli*, gyrase, which is made up of two GyrA subunits and two GyrB subunits, is the major target of (fluoro) quinolones. Topoisomerase IV, consisting of two ParC and two ParE subunits, is the secondary target (Weidlich and Klostermeier, 2020). Mutations in the genes for DNA gyrase (*gyrA*) and topoisomerase IV (*parC*) are the main cause of resistance to these drugs, also known as the quinolone resistance-determining regions. Mutations in the genes *gyrB* and *parE* can also weaken the therapeutic effect of (fluoro) quinolones (Hastak et al., 2020; Zhao et al., 2021). Target site mutation is a potential strategy for combating drug resistance. The identification and development of a novel class of chemicals that inhibit the two bacterial target enzymes and stabilize the DNA cleavage complexes were described recently by Lapointe et al. (2021). More study into it is likely to lead to the development of novel antibiotics.

Drug accumulation and uptake can be reduced by altering the outer membrane permeability and/or active efflux. Research on the active efflux pump of *E. coli* reveals that there are mainly five transmembrane transporter protein families involved in the active efflux of antibiotics: ATP binding cassette, major facilitator super-family, resistance nodulation division, multi-drug and toxic compound extrusion, and small multi-drug resistance (Nolivos et al., 2019). The well-studied AcrAB-TolC efflux pump belongs to the transporter RND super-family, which can recognize a variety of antibiotics (tetracycline, chloramphenicol, β-lactam, quinolone, *etc*.) and block the transport channels of the membrane, thus reducing the therapeutic effect of antibiotics (Reuter et al., 2020). The development of efflux pump inhibitors and structural modifications of antibiotics could increase the sensitivity of pathogenic bacteria to antibiotics.

### 2.2 *B. Fragilis*

#### 2.2.1 The Antibiotic Resistance Mechanism of *B. Fragilis*

*B. fragilis* is a Gram-negative anaerobic bacillus that causes opportunistic infections in humans. *B. fragilis* accounts for only about 1% of the gastrointestinal tract flora, while it causes 60 to 90% of all anaerobic infection (Wexler, 2007). *B. fragilis*, the predominant anaerobes isolated from a female genital tract infection (Krivak et al., 2004) can also infect multiple anatomical sites, causing peritonitis, pelvic abscess, and TOA, with an associated mortality rate of over 19% (Yekani et al., 2020). At present, the antibiotics commonly used in the clinical treatment of *B. fragilis* infection include clindamycin, metronidazole, cephalosporins, the fourth generation of quinolones, and carbapenems. Antibiotic resistance in *B. fragilis* has become more common in recent years (Nagy et al., 2011; Kangaba et al., 2015; Ferløv-Schwensen et al., 2017; Kouhsari et al., 2019; Sóki et al., 2020). Multiple antibiotic-resistant strains have been detected in Europe (Urbán et al., 2015; Ferløv-Schwensen et al., 2017; Kierzkowska et al., 2019), the USA (Husain et al., 2013; Merchan et al., 2016), Japan (Nakamura et al., 2017), and China (Wang et al., 2020), posing a great challenge to the clinical treatment of *B. fragilis* infection.

Currently, *B. fragilis* shows resistance to almost all commonly used antibiotics to different degrees. Although the vast majority of *B. fragilis* remains susceptible to metronidazole with a resistance rate of approximately 1%, decreased susceptibility to metronidazole has been reported, with some reference laboratories reporting resistance rates as high as 7.5% (Seifert and Dalhoff, 2010; Treviño et al., 2012; Goldstein et al., 2018; Maraki et al., 2020b). Carbapenems are the second most effective antibiotics for the treatment of *B. fragilis* infections. The resistance rate to imipenem has been reported at no more than 4% in most countries and regions (Snydman et al., 2010; Sheikh et al., 2015; Yim et al., 2015). The resistance rate to cefoxitin and B-lactamase inhibitor combinations is less than 10% (Sheikh et al., 2015; Snydman et al., 2017), while *B. fragilis* is almost 100% resistant to ampicillin (Gao et al., 2019). Most countries and regions are now reporting moxifloxacin resistance rates of more than 10% for *B. fragilis*, and the resistance rates are increasing (Wybo et al., 2007; Lee et al., 2015). Clindamycin resistance rates Vary considerably among different countries or regions: 2.8% in Romania (Szekely et al., 2015), 22.7% in Argentina (Fernandez-Canigia et al., 2012), 48.9% in Taiwan (Wang et al., 2014), and 51% in Korea (Roh et al., 2009). Tetracycline resistance rates vary more among countries: 9.7% in Spain (Betriu et al., 2008), while 84.62% in China (Wang et al., 1998). A recent study explored the antibiotic resistance of 78 isolates of *B. fragilis* and found that penicillin G (100%) had the highest resistance rate, followed by tetracycline (74.4%), clindamycin (41%), and cefoxitin (38.5%). Only one isolate tested positive for imipenem resistance, but metronidazole was effective against all isolates (Jasemi et al., 2021).

#### 2.2.2 The Antibiotic Resistance Mechanism of *B. Fragilis* Resistance

Currently, the mechanism of metronidazole resistance of *B. fragilis* is mainly focused on the *nin* gene (**Table 2**) (Lofmark et al., 2005). *nim* genes are usually associated with an insertion sequence (IS). The mechanism of NIM protein has not been fully explained, while it is generally considered to work as nitroreductases that reduce the bactericidal effect of metronidazole by targeting the nitro group of nitroimidazoles. There are many reports of *nim* gene-induced resistance. Löfmark *et al*. found that *nim*-positive strains are more likely to be induced to high levels of metronidazole resistance (Leitsch et al., 2014). According to Leitsch *et al*., there is no direct link between *nim* gene expression and resistance level. To date, nine *nim* genes have been reported for *B. fragilis* (*nimA-H* and *nimJ*), and each genotype is highly specific for IS elements, including *nimA* plP417 IS1186, *nimB* chromosome IS1186, *nimC* plP419 IS1170, *nimD* plP421 IS1169, and *nimE* ISBf4 (Ghotaslou et al., 2018). Other mechanisms of resistance have also been reported, such as metronidazole efflux, RecA protein over-expression, deficiency of the ferrous transport protein FeoAB, and modifications by other organisms (Hermsen et al., 2005).

**Table 2:** The main mechanism of drug resistance in *B. fragilis*.

Mechanism	Related research	Related drugs
Target site mutation	Reduce the nitro group of nitroimidazoles: *nim* (Lofmark et al., 2005) *ermE, ermG* and *ermB1* (Shoemaker et al., 2001; Roberts, 2004) *gyrA* (Oh et al., 2001), *gyrB* (Ricci et al., 2004)	Metronidazole clindamycin quinolone
Production of hydrolase or modified enzyme	Degrade carbapenems: *cfiA* (Podglajen et al., 2001), conjugated to imipenem: PBP2Bfr (Ayala et al., 2005)	Carbapenem
Active efflux pump or ribosomal protection	*tetX* (Moore et al., 2005), *tetX1* (Bartha et al., 2011)	Tigecycline

The synthesis of class B metal-β-lactamases, encoded by the *cfiA* gene, is now the predominant determinant of carbapenem resistance in *B. fragilis*. It can degrade carbapenems, thereby rendering the bacteria resistant. According to Sóki *et al*., the *cfiA* gene could be activated and become extremely resistant to imipenem and meropenem when particular IS elements (IS613, IS614B, IS1186, and IS1187) were introduced into the upstream region of the gene. *CfiA* genes can also be activated by their promoters (Podglajen et al., 2001). Ayala *et al*. showed that the altered affinity for PBP2Bfr, which was conjugated to imipenem, could also be associated with imipenem resistance (Ayala et al., 2005).

Active drug efflux and mutations in the quinolone-resistance-determining region DNA gyrase component *gyrA* are the most common causes of quinolone resistance in *B. fragilis*. Oh *et al*. found that, among 31 isolates of *B. fragilis* resistant to all quinolones, 15 isolates had *gyrA* mutation and 16 isolates with high resistance lacked *gyrA* mutation, indicating the existence of other resistance mechanisms (Oh et al., 2001). Ricci *et al*. detected another mutation in *gyrB* in one laboratory strain and two clinical isolates (Ricci et al., 2004).

In anaerobic bacteria, clindamycin resistance is mainly caused by changes in ribosome target, active efflux, and the development of erythromycin-resistant methylase (Erm). The main *erm* genes for clindamycin resistance in *B. fragilis* include *ermE*, *ermG*, and *ermB1* (Shoemaker et al., 2001; Roberts, 2004). The resistance mechanisms of tetracyclines include ribosomal protection, exocytosis pump activity, and enzyme inactivation. It is mainly related to the production of ribosomal protection protein (*tetX*) (Moore et al., 2005) in *B. fragilis*. A Hungarian study showed the presence of *tetX* and *tetX1* genes in some isolates exhibiting elevated tigecycline MICs, which may be responsible for the resistance (Bartha et al., 2011).

### 2.3 Gram- Positive Anaerobic Cocci

#### 2.3.1 Antibiotic Resistance of Gram-Positive Anaerobic Cocci

Gram-positive anaerobic cocci (GPAC) are indigenous flora of the skin and mucosal surfaces of the mouth and upper respiratory tract, the gastrointestinal system, and the female genitourinary tract (Murdoch, 1998; Kononen et al., 2007; Brook, 2008). This heterogeneous group has gone through significant taxonomic changes. In the literature, GPAC has been described by various synonyms, such as “anaerobic coccus”, “anaerobic streptococcus”, and “*Peptococcus* and *Peptostreptococcus*” (Murphy and Frick, 2013). Infections involving GPAC are often polymicrobial. Of all isolated anaerobic bacteria from clinical specimens, GPAC account for approximately 25–30% (Japanese Society of Chemotherapy Committee on Guidelines for Treatment of Anaerobic, i., and Japanese Association for Anaerobic Infections, R, 2011; Murphy and Frick, 2013). This part will discuss the GPAC species associated with the pathogenesis of the female reproductive tract infection. Among GPAC, *Peptostreptococcus* spp. is one of the most pathogenic bacteria. Statistics show that the prevalence of *Peptostreptococcus* spp. in PID patients appeared at a steady rate of 7 to 8% from 1999 to 2001, indicating that they were the most common pathogens among anaerobes (Skapinyecz et al., 2003).

Until recently, the data on the antimicrobial susceptibility test of the different species of GPAC is frequently based on GPAC in general. Increasing resistance trends have been reported in recent years globally, and clinical failures in patients receiving inappropriate treatments have been reported (Brazier et al., 2003; Brazier et al., 2008; Veloo et al., 2011; Goldstein et al., 2017; Goldstein et al., 2020; Maraki et al., 2020a). In the United States, according to the drug susceptibility test of anaerobes in adults collected between 2007 and 2012 by Hastey *et al*., there were substantial increases in resistance to ampicillin–sulbactam (1–9%), cefoxitin (0–3%), moxifloxacin (11–20%), and ertapenem (0–9%) in the GPAC (Hastey et al., 2016). In a study of 299 GPAC strains from 10 European nations, Brazier *et al*. discovered that tetracycline resistance accounted for 41.6% of the total resistance among GPAC, with erythromycin resistance accounting for 27.4%. Overall, 7% of the strains were resistant to penicillin and/or clindamycin (Brazier et al., 2008), even though it is widely accepted that GPAC species retain much greater susceptibility to cephalosporins, carbapenems, chloramphenicol, and β-lactam/β-lactamase inhibitors. Generally speaking, GPAC exhibit varying resistance to penicillins (2–25%), clindamycin (3–24%), and metronidazole (3–15%) while having a much greater susceptibility to β-lactam/β-lactamase inhibitors, cephalosporins, carbapenems, and chloramphenicol (Hastey et al., 2016; Yunoki et al., 2017; Byun et al., 2019; Cobo et al., 2019; Badri et al., 2019; Forbes et al., 2021; Guerin et al., 2021).

#### 2.3.2 The antibiotic resistance mechanism of GPAC

GPAC-acquired antibiotic resistance mainly includes the target site mutation, enzymatic hydrolysis, and efflux pump system (**Table 3**). One of the primary causes of GPAC resistance is the process of target site mutation. By altering or modifying the target site of antibiotic medicines or by making the target site mutated, GPAC can make antibiotics insensitive and develop drug resistance—for instance, metronidazole, a 5-nitroimidazole, has long been a favored antibiotic for treating serious anaerobic infections (Hernandez Ceruelos et al., 2019). Certain GPAC species have developed resistance—for example, *nim* genes in *Peptostreptococcus* spp., possibly located on mobile genetic elements, encode nitroimidazole reductases responsible for drug inactivation (Alauzet et al., 2019; Thomas and Gwenin, 2021). Until now, 11 *nim* genes (*nimA* to *nimK*) have been identified, but only the *nimB* gene has been found in the chromosome of certain GPAC isolates (*Peptostreptococcus anaerobius*, *Peptostreptococcus prevotii*, *Parvimonas micra*, and *Finegoldia magna*) (Theron et al., 2004; Alauzet et al., 2019; Guerin et al., 2020).

**Table 3:** The main mechanism of drug resistance in GPAC.

Mechanism	Related research	Related drugs
Reduced affinity to target molecule	PBP alterations (Gajdacs et al., 2017)	β-lactams
target site mutation	Methylation of the 23S rRNA: *ermA*, *ermB*, *ermF*, and *ermTR* (Reig et al., 2001; Guerin et al., 2020; Guerin et al., 2021) Nitroimidazole reductase: *nimB* (Theron et al., 2004)	Macrolides Metronidazole
Active efflux pump or ribosomal protection	*tetK*, *tetL*, *tetM*, *tetO* (Roberts and Hillier, 1990; Chopra and Roberts, 2001)	Tetracyclines

Macrolides inhibit bacterial protein synthesis by binding reversibly to the 23S rRNA at a site near the peptidyl transferase center of the 50S ribosomal subunit. The *erm* family genes encode adenine-specific N-methyltransferases that methylate the 23S rRNA to prevent antibiotic binding. It is noteworthy that the genetic basis of resistance to macrolides–lincosamides–streptogramins in GPAC is not fully elucidated; only three studies have looked into the molecular basis: *ermA*, *ermTR*, *ermB*, and *ermF* have been identified in GPAC strains (Reig et al., 2001; Guerin et al., 2020; Guerin et al., 2021). A β-lactam/β-lactamase inhibitor combination is widely used in the treatment of anaerobic infections. In a recent study, two *Parvimonas micra* strains were found to be resistant to penicillin (Brazier et al., 2008). The penicillin-resistant strains did not produce b-lactamase, and penicillin-binding protein (PBP) modifications appear to account for most β-lactam resistance (Reig and Baquero, 1994; Hecht, 2006; Gajdacs et al., 2017).

Tetracycline resistance is so widespread in anaerobic isolates (>40% of GPAC) that it is no longer recommended clinically. Drug efflux (*tetK-L*) and ribosome protection (*tetM* and *tetO*) are two tetracycline resistance mechanisms reported in GPAC (Roberts and Hillier, 1990; Chopra and Roberts, 2001; Grossman, 2016). TetM and TetO are cytoplasmic proteins that protect the ribosomes from the action of tetracycline, doxycycline, and minocycline (Burdett, 1991; Trieber et al., 1998; Gao et al., 2018). *tetK* and *tetL* genes code for membrane-associated proteins which export tetracycline from the cell, thus reducing the intracellular drug concentration and protecting the ribosomes within the cell.

## 3 Future perspective: New direction for antibiotic resistance

Traditional strategies for developing new antibiotics include screening for new target drugs that inhibit bacterial metabolism and survival pathways or developing mixed compounds that are mechanistically more effective than their components (Rusu and Buta, 2021). In basic research and preclinical studies, there are novel methodologies that could provide therapy alternatives in the near future. Innovative applications of clustered regularly interspaced short palindromic repeats (CRISPR), nanoparticles (NPs), phage treatment, and antimicrobial peptides (AMPs), and pathogen-specific monoclonal antibodies have the potential to transform antimicrobial therapy.

Bacteria have an immune system defending them from phages that may introduce foreign genetic material. This immune system consists of restriction enzymes, toxin–antitoxin systems, and the CRISPR–Cas system (Bondy-Denomy et al., 2015; Watson et al., 2021). Citorik et al. have developed RNA-guided nucleases targeting antibiotic resistance and virulence determinants in *E. coli*. Specific DNA sequences of the resistance genes blaSHV-18 and blaNDM-1 are delivered efficiently to microbial populations using bacteriophage or bacteria-carrying plasmids transmissible by conjugation. According to the researchers, the number of *E. coli* strains to carry the targeted resistance gene was remarkably reduced either chromosomally or plasmidic (Citorik et al., 2014). The ability to silence target genes on bacterial chromosomes has been confirmed in many other multi-drug resistant strains (*Staphylococcus aureus*, *Streptococcus pneumoniae*, and *Salmonella*) (Jiang et al., 2013; Bikard et al., 2014; Gomaa et al., 2014; Gholizadeh et al., 2020). The CRISPR–Cas genome editing technique has quickly proved that it is effective at targeting antibiotic resistance genes with great sensitivity and specificity. This demonstrates the CRISPR–Cas system’s utility in the development of novel antimicrobial drugs (Greene, 2018).

Bacteriophages are bacterial viruses that are widely spread in nature and play an important role in bacterial ecology. Though the first paper on phage treatment was published in the 1920s (Summers, 2001), it was not until the rise of antibiotic-resistant infections that the role of phages once again attracted attention (Hill et al., 2018). Phage treatment involves infecting and lysing a specific bacterial population or inactivating resistance genes with bacteriophages. There have been many successful cases of phage treatment of multi-drug resistance in humans (Leitner et al., 2017; Jennes et al., 2017; Jault et al., 2019; Ooi et al., 2019). A recent and representative study of phage therapy was reported in a diabetic patient, with necrotizing pancreatitis complicated by a multi-drug-resistant *A. baumannii* infection, who failed to respond to antibiotic treatment. The elimination of the infection and improvement of the patients’ conditions was shown after phage therapy (Schooley et al., 2017). Bacteriophages might theoretically be developed to target any bacterial species with minimum collateral microbiome modification due to their abundance, genetic diversity, and specificity (Furfaro et al., 2018).

NPs are metals or metal oxides that fight bacteria by disrupting the cell membrane, releasing reactive oxygen species, and causing harm to intracellular contents. Some nanomaterials act as carriers, and they can prevent biofilm formation and deliver drugs to infection areas with excellent penetration and retention (Baptista et al., 2018). The use of NPs in combination with antibiotics has been shown to operate synergistically, reducing antibiotic and NP doses, achieving a high local concentration, or even reversing antibiotic resistance (Vestergaard et al., 2019; Chavan et al., 2020; Motelica et al., 2021).

AMPs refer to polyamides that can be made on a contemporary chemical peptide synthesizer and be able to exert antimicrobial activity (Fodor et al., 2020). AMPs are a kind of innate immunological molecule that has both microbicidal and immunomodulatory properties, acting as the first line of defense against pathogenic invasion. Many efforts are being made to find particular AMPs with broad-spectrum antimicrobial efficacy against pathogenic bacteria. Zhao et al. discovered a new 34-amino-acid residue, cathelicidin, with significant microbicidal action against a variety of gram-negative and gram-positive bacteria (Zhao et al., 2018). Besides this, AMPs in combination with conventional antibiotics can be effective in lowering resistance through synergistic effects (Sakoulas et al., 2012; Amani et al., 2015). Pathogen-specific monoclonal antibodies can also target bacterial infections with little danger of resistance or disruption of the microbiome (DiGiandomenico and Sellman, 2015). Techniques for isolating immunoglobulin directly from human B cells from infected individuals or after vaccination have also been developed (Moffett et al., 2019). By far, there is no preclinical experiment or clinical trials of these novel drugs designed to cure TOA, and this will be a promising research direction.

## 4 Conclusion

TOA caused by polymicrobial pathogens has become one of the main safety issues threatening female health. Early initiation of broad-spectrum antibiotics is the standard treatment of TOA. However, multi-drug resistant bacteria have been isolated all over the world as a result of widespread or irregular antibiotic usage, posing a threat to antibiotic efficacy. This article summarizes the main pathogenic bacteria of TOA and introduces the drug resistance mechanism of the three main typical pathogens isolated from TOA. In summary, the current resistance mechanisms of TOA-pathogenic bacteria mainly include the mechanism of production of hydrolase or modified enzymes, reducing the permeability of the cell membrane, the efflux pump mechanism, ribosomal protection, and the mechanism of target site mutation. Facing the increasingly serious problem of antimicrobial resistance, we need to focus on improving the research on the resistance of TOA pathogens from multiple aspects. On the one hand, although the specific mechanism of female genital infection remains to be studied in depth, we can choose the relatively sensitive antibiotics to prevent and treat patients with TOA. On the other hand, there is an urgent need for new approaches that adopt different bactericidal mechanisms to avoid drug resistance and provide the capability to only target harmful bacteria with minimal effect on patients and other beneficial bacteria. Scientists have made breakthroughs in basic researches and preclinical studies; there are novel methodologies that could provide therapy alternatives in the near future.

## Author Contributions

HT drafted the manuscript for publication. HZ participated in writing the chapter on *B. fragilis* resistance. HZ and RZ reviewed and revised the manuscript. All authors contributed to the article and approved the submitted version.

## Conflict of Interest

The authors declare that the research was conducted in the absence of any commercial or financial relationships that could be construed as a potential conflict of interest.

## Publisher’s Note

All claims expressed in this article are solely those of the authors and do not necessarily represent those of their affiliated organizations, or those of the publisher, the editors and the reviewers. Any product that may be evaluated in this article, or claim that may be made by its manufacturer, is not guaranteed or endorsed by the publisher.
